# *Klebsiella* spp. cause severe and fatal disease in Mozambican children: antimicrobial resistance profile and molecular characterization

**DOI:** 10.1186/s12879-021-06245-x

**Published:** 2021-06-05

**Authors:** Arsénia J. Massinga, Marcelino Garrine, Augusto Messa, Nélio A. Nobela, Nadia Boisen, Sergio Massora, Anélsio Cossa, Rosauro Varo, António Sitoe, Juan Carlos Hurtado, Jaume Ordi, Hélio Mucavele, Tacilta Nhampossa, Robert F. Breiman, Cynthia G. Whitney, Dianna M. Blau, Quique Bassat, Inácio Mandomando

**Affiliations:** 1 Centro de Investigação em Saúde de Manhiça (CISM), Maputo, Mozambique; 2 Global Health and Tropical Medicine, Instituto de Higiene e Medicina Tropical, Universidade Nova de Lisboa (IHMT, UNL), Lisbon, Portugal; 3 Department of Bacteria, Parasites and Fungi, Statens Serum Institut, Copenhagen, Denmark; 4 ISGlobal, Hospital Clínic-Universitat de Barcelona, Barcelona, Spain; 5 Instituto Nacional de Saúde (INS), Ministério da Saúde, Maputo, Mozambique; 6 Emory Global Health Institute, Emory University, Atlanta, GA USA; 7 Center for Global Health, Centers for Disease Control and Prevention, Atlanta, GA USA; 8 ICREA, Pg. Lluís Companys 23, 08010 Barcelona, Spain; 9 Consorcio de Investigación Biomédica en Red de Epidemiología y Salud Pública (CIBERESP), Madrid, Spain

**Keywords:** *Hypermucoviscosity*, Bacteremia, ESBL genes, Hypervirulence, CTX-M-15, Mozambique, SPATEs

## Abstract

**Background:**

*Klebsiella* spp. are important pathogens associated with bacteremia among admitted children and is among the leading cause of death in children < 5 years in postmortem studies, supporting a larger role than previously considered in childhood mortality. Herein, we compared the antimicrobial susceptibility, mechanisms of resistance, and the virulence profile of *Klebsiella* spp. from admitted and postmortem children.

**Methods:**

Antimicrobial susceptibility and virulence factors of *Klebsiella* spp. recovered from blood samples collected upon admission to the hospital (*n =* 88) and postmortem blood (*n =* 23) from children < 5 years were assessed by disk diffusion and multiplex PCR.

**Results:**

*Klebsiella* isolates from postmortem blood were likely to be ceftriaxone resistant (69.6%, 16/23 vs. 48.9%, 43/88, *p* = 0.045) or extended-spectrum β-lactamase (ESBL) producers (60.9%, 14/23 vs. 25%, 22/88, *p =* 0.001) compared to those from admitted children. bla_CTX-M-15_ was the most frequent ESBL gene: 65.3%, 9/14 in postmortem isolates and 22.7% (5/22) from admitted children. We found higher frequency of genes associated with hypermucoviscosity phenotype and invasin in postmortem isolates than those from admitted children: rmpA (30.4%; 7/23 vs. 9.1%, 8/88, *p =* 0.011), *wzi-K1* (34.7%; 8/23 vs. 8%; 7/88, *p =* 0.002) and traT (60.8%; 14/23 vs. 10.2%; 9/88, *p* < 0.0001), respectively. Additionally, serine protease auto-transporters of Enterobacteriaceae were detected from 1.8% (*pic*) to 12.6% (*pet)* among all isolates. *Klebsiella* case fatality rate was 30.7% (23/75).

**Conclusion:**

Multidrug resistant *Klebsiella* spp. harboring genes associated with hypermucoviscosity phenotype has emerged in Mozambique causing invasive fatal disease in children; highlighting the urgent need for prompt diagnosis, appropriate treatment and effective preventive measures for infection control.

**Supplementary Information:**

The online version contains supplementary material available at 10.1186/s12879-021-06245-x.

## Background

*Klebsiella* spp. are among the most frequently reported pathogens associated with opportunistic infections in immunocompromised individuals and patients hospitalized for prolonged periods [1], and commonly implicated in nosocomial urinary tract infections and sepsis in infants from sub-Saharan Africa [2, 3]. Given the importance of *Klebsiella* spp. in nosocomial infection and the continuous increase and spread of community-acquired multi-drug resistant (MDR) *Klebsiella pneumoniae*, including Extended-Spectrum β-Lactamase (ESBL) producers, the associated high mortality is causing a grave concern [2, 4].

A new *K. pneumoniae*, called hypervirulent (hv*Kp*) is a global public health concern because of its invasiveness (e.g. causing pyogenic liver abscess, bacteremia, pneumonia), the capacity to form metastasis and for being more virulent than the classical *K. pneumoniae* (c*Kp*), affecting healthy individuals in the community settings [5, 6]. The hv*Kp* can be distinguished from the classical ones by specific biomarkers, namely, the presence of siderophores (aerobactin [*iuc*] system), hypermucoviscosity (encoded by *rmpA*, *rmpA2*) and salmochelin (*iro*) [6–9].

The virulence genes of both cKp and hvKp includes: siderophores for the iron acquisition (e.g. enterobactin (*ent*), adhesins for adherence (eg. *fimH*, *mrkD*), invasins for invasion and escape of the immune system defence (eg. *traT*), and protectins (eg. *magA* responsible for the hypermucoviscous phenotype) [10–13].

Recently, postmortem studies conducted in urban (Maputo city) and rural (Manhiça District) Mozambique to ascertain causes of death in stillbirths and young children using minimally invasive tissue sampling (MITS) approaches found *Klebsiella* spp. to be among the top pathogens assigned in the chain of events leading to death [14–16].

Data on the molecular epidemiology and characterization of *Klebsiella* spp. are scarce in Africa including Mozambique [17–19]. However, data from an urban hospital in the city of Beira, central Mozambique showed high frequency (100% of isolates) of ESBL-producing *K. pneumoniae* among hospitalized children with urinary tract infections [20]. Additionally, in our previous work we documented the occurrence of community-acquired *Klebsiella* spp. strains harboring *bla*_*CTX-M-15*_ among children living in the Manhiça District, southern Mozambique [21, 22]. Herein, we described and compared the molecular characterization, antimicrobial susceptibility of *Klebsiella* spp. isolates recovered from bloodstream infections and postmortem blood specimens collected in children under 5 years of age and we characterize the MDR associated *Klebsiella* spp. from admitted children with bloodstream infections who have died. In this prospective study, we documented the occurrence of hypermucoviscous, multidrug-resistant *Klebsiella* spp. with an ESBL-producing phenotype, harboring *bla*_*CTX*-*M-15*_, and causing invasive disease associated with poor outcomes in infants and young children in a rural hospital in Southern Mozambique.

## Methods

### Clinical isolates and study area

We analyzed 111 *Klebsiella* spp. isolates recovered from bloodstream infection (*n =* 88) of children under 5 years old admitted at the Manhiça District Hospital (January 2001 and December 2019) and from postmortem blood specimens (*n =* 23) of deceased children under 5 years old enrolled in the ongoing Child Health and Mortality Prevention Surveillance (CHAMPS) project (December 2016 and December 2019) [23]. The CHAMPS Network was established in seven countries in south Asia and sub-Saharan Africa including Mozambique (Manhiça site) to collect standardized, population-based, longitudinal data on mortality among children under 5 years and stillbirths, to improve the accuracy of causes of death determination using a novel approach of minimally invasive tissue sampling (MITS) [24].

In Mozambique, CHAMPS is being conducted by the Centro de Investigação em Saúde de Manhiça (CISM) in the district of Manhiça, a rural area of Maputo Province in southern Mozambique, with an estimated population of approximately 201,845 inhabitants. In this site, a continuous demographic surveillance system (DSS) has been running since 1996 with regular update of vital events; and currently covering the entire district of Manhiça [25].

In addition to the DSS, CISM has been conducting morbidity surveillance which includes invasive bacterial disease surveillance in the pediatric population (children < 15 years of age) at the Manhiça District Hospital (MDH) (a 150 beds referral district hospital of the Manhiça district) and other 5 peripheral health facilities since January 1997, documenting all outpatients and inpatients visits using standard questionnaires. Blood cultures were routinely performed as part of the clinical practice upon hospital admission for all children under 2 years old and for older children under 15 years with axillary temperature ≥ 39 °C or who meet, according to the admitting clinician, specific severity criteria [4, 26]. Bacterial cultures were performed at the CISM Microbiology laboratory for *Klebsiella* spp. and other bacterial isolation and identification using conventional microbiology and biochemical methods [4]. Clinically relevant isolates were stored at − 70 °C in a biobank (in a STGG medium – 2% of Skim milk + 3% tryptic soy broth + 0.5% glucose + 10% glycerol) for further characterization [27]. *Klebsiella* bacteremia was defined as *Klebsiella* positive blood culture collected from children upon admission at the MDH.

Under the health demographic surveillance system (HDSS) platform, CISM has been conducting a mortality surveillance in the context of the CHAMPS network, since 2016. CHAMPS uses the MITS approach on deaths reported from the community or health facility, to track the definitive/immediate causes of deaths in stillbirths and children under 5 years old, as described elsewhere [14, 23]. Tissue samples, including blood and cerebrospinal fluid (CSF) are investigated using conventional microbiology and molecular techniques for pathogens detection, including *Klebsiella* [23, 28, 29]. The causes of death were determined by a panel of experts that comprises at least 1 of each of the following: clinician (eg, pediatricians, neonatologists, and obstetricians), pathologist, epidemiologist, and microbiologist. The Panelists receive all available information from linked maternal data, child clinical data, individual demographic data, verbal autopsy, microbiology, molecular testing, clinical diagnostics (human immunodeficiency virus [HIV], tuberculosis, malaria), photographs of the deceased from the MITS procedure, and histopathology findings in the form of a determination of cause of death package and assign the underlying, antemortem, and immediate (and other contributing) causes of death [14]. Postmortem *Klebsiella* is defined as *Klebsiella* isolates recovered from blood samples collected through MITS in deceased children enrolled in the CHAMPS.

### Antimicrobial susceptibility testing and empirical therapy

The antimicrobial susceptibility phenotypes for ampicillin (AMP), amikacin (AMK), amoxicillin-clavulanic acid (AUG), piperacillin-tazobactam (TZP), cefuroxime (CXM), ceftriaxone (CRO), ceftazidime (CAZ), aztreonam (ATM), ertapenem (ETP), imipenem (IPM), meropenem (MEM), nalidixic acid (NA), ciprofloxacin (CIP), chloramphenicol (CHL), tobramycin (TOB), gentamicin (GEN), tetracycline (TET) and trimethoprim-sulfamethoxazole (SXT) were determined for all isolates by conventional disk diffusion on Mueller Hinton agar (BD, Heidelberg, Germany) using commercially available disks (BD, Heidelberg, Germany). The selection of the antibiotics tested was done according to their use for local empirical treatment and those recommended by the Clinical Laboratory Standard Institute (CLSI) guidelines [30]. The interpretative category of resistance was determined according to the CLSI guidelines (29th Edition) [30]. All isolates resistant to three or more unrelated families of antibiotics were defined as MDR [31]. In the MDH, children are treated empirically with ampicillin or ampicillin + gentamicin and amikacin or ceftriaxone.

### Detection of ESBL phenotype and genotype

All ceftriaxone, aztreonam or ceftazidime resistant isolates were then screened for ESBL production by disk synergy assay with the addition of a beta-lactamase inhibitor disc, as previously described [32]. Briefly, AMP, CAZ, and CRO disk were placed 2 cm distant and AUG disk was placed on the center closer to the other disks on Muller-Hinton agar plate inoculated with 0.5McF of *Klebsiella* isolates. The tests were considered positive if lightly growth-inhibitory zone (phantom zone) was observed between the disks. Isolates with ESBL phenotype were assessed by a multiplex polymerase chain reaction (PCR) for *bla*_*CTX-M*_*, bla*_*TEM*_*, bla*_*SHV*_ genes, using universal primers (Table 1). The multiplex PCR was performed in 25 μL reaction volumes, including 0.5 μM of each primer, 12,5 μL of 2X master mix (Fermentas, Thermo Scientific, MA, USA), 3 μL of genomic DNA and nuclease free water. Cycling conditions included an initial denaturation step of 5 min at 95 °C, followed by 30 amplification cycles (denaturation at 94 °C for 1 min, annealing at 57 °C for 1 min and elongation at 72 °C for 2 min) and a final extension step at 72 °C for 10 min.

**Table 1:** Primers used for PCR assays

Target	Oligonucleotide sequence (5′–3′)	Annealing temperature (°C)	Expected size (bp)	Reference
*bla*_*CTX-M*_	F: CGATGTGCAGTACCAGTAA	57	579	[33]
	R: TTAGTGACCAGAATCAGCGG			
*bla*_*SHV*_	F: ATGCGTTATATTCGCCTGTG	57	841	[34]
	R: TTAGCGTTGCCAGTGCTCG			
*bla*_*TEM*_	F:ACGCTCAGTGGAACGAAAAC	57	1150	[33]
	R: ATTCTTGAAGACGAAAGGGC			
*mrkD*	F: CCACCAACTATTCCCTCGAA	57	240	[11]
	R:ATGGAACCCACATCGACATT			
*rmpA*	F: ACTGGGCTACCTCTGCTTCA	57	537	[35]
	F: CTTGCATGAGCCATCTTTCA			
*wzi-K1*	F: GGTGCTCTTTACATCATTGC	57	1282	[35]
	R: GCAATGGCCATTTGCGTTAG			
*entB*	F: ATTTCCTCAACTTCTGGGGC	57	371	[11]
	R: AGCATCGGTGGCGGTGGTCA			
*ycfM*	F: ATCAGCAGTCGGGTCAGC	57	160	[11]
	R: CTTCTCCAGCATTCAGCG			
*iutA*	F: GGCTGGACATCATGGGAACTGG	57	300	[36]
	R: CGTCGGGAACGGGTAGAATCG			
*fyuA*	F: GCGACGGGAAGCGATGATTTA	57	547	[11]
	R: TAAATGCCAGGTCAGGTCACT			
*hlyA*	F: AACAAGGATAAGCACTGTTCTGGCT	63	1177	[11]
	R: ACCATATAAGCGGTCATTCCCGTCA			
*traT*	F: GGTGTGGTGCGATGAGCACAG	63	290	[36]
	R: CACGGTTCAGCCATCCCTGAG			
*hlyC*	F: AGGTTCTTGGGCATGTATCCT	55	556	[37]
	R: TTGCTTTGCAGACTGCAGTGT			
*fimH*	F: ATGAACGCCTGGTCCTTTGC	55	688	[11]
	R: GCTGAACGCCTATCCCCTGC			
*pet*	F: GGCACAGAATAAAGGGGTGTTT	58	302	[38]
	R: CCTCTTGTTTCCACGACATAC			
*piC*	F: ACTGGATCTTAAGGCTCAGGAT	58	572	[38]
	R: GACTTAATGTCACTGTTCAGCG			
*sat*	F: TCAGAAGCTCAGCGAATCATTG	58	932	[39]
	R: CCATTATCACCAGTAAAACGCACC			
*sepA*	F: GCAGTGGAAATATGATGCGGC	58	794	[38]
	R: TTGTTCAGATCGGAGAAGAACG			
*sigA*	F: CCGACTTCTCACTTTCTCCCG	58	430	[39]
	R: CCATCCAGCTGCATAGTGTTTG			

PCR amplicons were visualised on 2% ultrapure agarose gel (Invitrogen, CA, USA) stained with 25 μM ethidium bromide solution (Invitrogen, CA, USA).

Amplicons of the expected size, for *bla*_*CTX-M*_*, bla*_*TEM*_*, bla*_*SHV*_ genes were purified using QIAquick PCR purification kit (QIAGEN, Hilden, Germany), and shipped to a commercial sequencing services company (Macrogen Europe BV, Amsterdam, the Netherlands) for Sanger sequencing in both directions using the same sets of primers for each gene (Table 1). Sequence analysis and alignments were performed using the BioEdit software (v7.2.5) and the BLAST Program (http://blast.ncbi.nlm.nih.gov/Blast.cgi), which was also used to determine the allele correspondent for each gene (*bla*_*CTX-M*_*, bla*_*TEM*_*, bla*_*SHV*_).

### Detection of virulence genes

The virulence genes associated with protectins and invasins (*rmpA*, *wzi-K1,* and *traT*), adhesins (*fimH*, *mrkD,* and *ycfM*), toxins (*hylA* and *hylC*) and siderophores (*iutA*, *fyuA,* and *entB*) were assessed by 4 multiplexed PCRs for each group of genes, optimized in this study and using primers previously described (Table 1). All genes, except those encoding Serine Protease Auto-transporters of *Enterobacteriaceae* (SPATEs), were amplified in 25 μL reaction volumes, including 0.5 μM of each primer, 12,5 μL of 2X master mix (PCR Master Mix 2x, Thermo Scientific, MA, USA), 3 μL of genomic DNA and nuclease free water. The cycling conditions included an initial denaturation step at 95 °C for 2 min, followed by 35 amplification cycles with denaturation at 94 °C for 30 s, annealing for 1.5 min (temperatures are described in Table 1) an elongation at 72 °C for 1.5 min and a final extension step at 72 °C for 10 min.

The detection of SPATEs genes (*sat*, *sepA*, *sigA*, *piC,* and *pet*) were assessed by a multiplex PCR previously described [39]. Briefly the genes were amplified in 25 μL reaction volume, consisting of 25 μM of each primer, 12.5 μL of the QIAGEN master mix (QIAGEN multiplex PCR kit; QIAGEN, Courtaboeuf, France), 2.5 μL of Q-solution (QIAGEN, Courtaboeuf, France) and 3 μL of genomic DNA.

One PCR product of the expected size per SPATE gene (*sat*, *sepA*, *sigA*, *piC,* and *pet*) was also shipped for Sanger sequencing with the corresponding sets of primers (described in Table 1) for confirmation.

### Hypermucoviscous phenotype

To verify the concordance with the genotype and the expression of the hypermucoviscous phenotype, all *rmpA* and *wzi-K1* positive isolates were tested by string tests as previously described elsewhere [8].

### Statistical analysis

PCR and antibiotic susceptibility data were entered in an Excel spreadsheet and checked for consistency by an independent laboratory technician. Data were analyzed using Stata software version 14.0 (STATA Corporation, College Station, Tx, USA). Frequencies and proportions were used to describe categorical variables, and differences of proportions were compared using the chi-square or Fisher’s exact test where appropriate, with a *p-*value of < 0.05 being considered significant.

Surviving children or Srv. Bacteremia were defined as children hospitalized at the MDH who tested positive for *Klebsiella* spp. in blood culture upon admission and that were alive at discharge (*n =* 41) and dead children or Dth. Bacteremia were defined as children hospitalized at the MDH who tested positive for *Klebsiella* spp. in blood culture upon admission who died and had clinical information available *n =* 23).

## Results

### Description of studied isolates

**Fig. 1:**
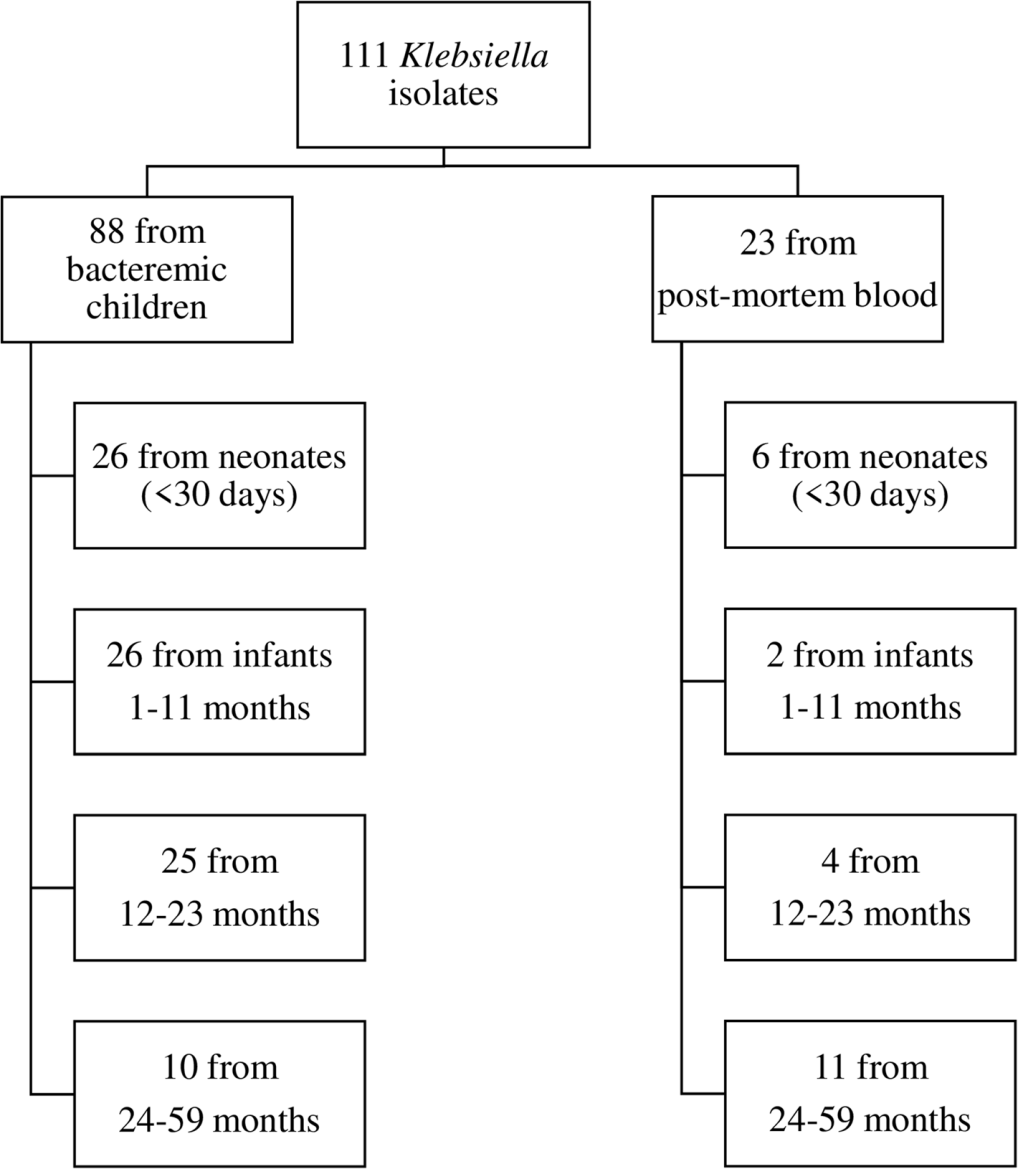
Demographic characteristics of the children from whom the isolates were recovered

From the study period (January 2000 – December 2019), 52,968 children younger than 5 years were admitted to the hospital in the context of invasive bacterial disease surveillance and had blood culture collected, yielding a positivity rate of 8.1% (*n =* 4270) for any bacteria. *Klebsiella* spp. accounted for 2.1% (88/4270) of the total positive; in addition, between December 2016 and December 2019, 226 deceased children were enrolled in the CHAMPS, and *Klebsiella* spp. were isolated in 23 blood postmortem specimens. In general, 72.7% (64/88) and 87% (20/23) were *K. pneumoniae,* 5.7% (5/88) and 13% (3/23) were *K. oxytoca*, from admitted children and post-mortem blood, respectively, and the remaining 21.6% (19/88) were classified as *Klebsiella* spp. Most isolates from admitted children and postmortem isolates were recovered from males, 55.7% (49/88) and 60.9% (14/23), respectively. *Klebisella* isolates were also recovered from neonates and children between 12 and 23 months (29.5%, 26/88) upon admission and from postmortem children between 23 and 59 months (47.8%, 11/23) (Fig. 1).

### Antimicrobial susceptibility and ESBL genes of *Klebsiella spp.*

Overall, resistance ranged from 51.4% (57/111) for amoxicillin-clavulanic acid, 53.1% (54/111) for ceftriaxone, 54.1% (60/111) for gentamicin to 92.7% (103/111) for ampicillin, with 66.7% (74/111) of isolates being MDR. Ceftriaxone resistance was more frequent among isolates recovered from postmortem compared to those from admitted children (69.6%, 16/23 vs. 48.9%, 43/88, *p* = 0.045). Eight percent (7/88) of isolates from admitted children had reduced susceptibility to meropenem, while 34.8% (8/23) and 13% (3/23) of the postmortem isolates showed full resistance to ertapenem and meropenem, respectively (Table 2), and 30.4% (7/23) of postmortem isolates had intermediate resistance to ertapenem.

**Table 2:** Antimicrobial resistance profile of *Klebsiella* isolates recovered from blood samples collected upon admission and postmortem blood samples

	On admission *n =* 88(%)	Postmortem *n =* 23(%)	*P* value
**AMP**	82 (93.1)	21 (91.3)	1
**AMK**	2 (2.3)	0	0.462
**AUG**	44 (50)	13 (56.5)	0.607
**TZP**	1 (1.1)	1 (4.3)	0.321
**CXM**	43 (48.9)	15 (65.2)	0.162
**CRO**	43 (48.9)	16 (69.6)	0.045
**CAZ**	18 (20.4)	15 (65.2)	0.001
**ATM**	42 (47.7)	12 (52.2)	0.526
**ETP**	0	8 (34.8)	< 0.0001
**IPM**	0	0	
**MEM**	0	3 (13)	0.009
**NA**	9 (10.2)	4 (17.4)	0.464
**CIP**	10 (11.4)	9 (39.1)	0.012
**CHL**	44 (50)	6 (26.1)	0.04
**GEN**	44 (50)	16 (69.6)	0.094
**TOB**	37 (42.1)	14 (60.9)	0.112
**TET**	30 (34.1)	6 (26.1)	0.521
**SxT**	68 (77.3)	11 (47.8)	0.233
**MDR**	58 (65.9)	16 (69.6)	0.741
**ESBL**	22 (25)	14 (60.9)	0.001

*AMP* Ampicillin, *AMK* Amikacin, *AUG* Amoxicillin-clavulanic acid, *TZP* Piperacillin-tazobactam, *CXM* Cefuroxime, *CRO* Ceftriaxone, *CAZ* Ceftazidime, *ATM* Aztreonam, *ETP* Ertapenem, *IPM* Imipenem, *MEM* Meropenem, *NA* Nalidixic acid, *CIP* Ciprofloxacin, *CHL* Chloramphenicol, *GEN* Gentamicin, *TOB* Tobramycin, *TET* Tetracycline, *SxT* Trimethoprim- Sulfamethoxazole, *MDR* Multi-drug resistant (defined when isolates are resistant ≥3 unrelated antibiotic groups), ESBL – Extended-spectrum β-lactamase phenotype

ESBL phenotype was higher among postmortem isolates (60.9%, 14/23) compared to those from admitted children (25%, 22/88), *p* = 0.001. Among beta-lactamase genes detected in postmortem isolates and in isolates from admitted children, respectively, *bla*_*CTX-M*_ accounted for 71.4% (10/14) and 41% (9/22), *p* = 0.075, *bla*_*SHV*_ accounted for 42.8% (6/14) and 27.2% (6/22), *p* = 0.33, and *bla*_*TEM*_ accounted for 57.1% (8/14) and 9.1% (2/22), *p* = 0.002. *bla*_*CTX-M-15*_ was found in 90% (9/10) of postmortem *bla*_*CTX-M*_ isolates and 55.6% (5/9) of *bla*_*CTX-M*_ isolates from admitted children, whereas *bla*_*SHV-1*_ was detected in 66.7% (4/6) and 50% (3/6) of postmortem and *bla*_*SHV*_ isolates from admitted children, respectively. All *bla*_*TEM*_ isolates were *TEM-1* variant.

### Virulence profile of *Klebsiella* isolates

Genes associated with the hypermuscoviscous phenotype or hypervirulence were more commonly found in the postmortem isolates compared to isolates from admitted children (Table 3): *wzi-K1* (34.7%; 8/23 vs. 8%; 7/88, *p* = 0.002), *rmpA* (30.4%; 7/23 vs. 9.1%; 8/88, *p* = 0.011), and invasins, *traT* (60.8%, 14/23 vs. 10.2%, 9/88, *p* < 0.0001), respectively. Those three genes were simultaneously detected among 17.3% (4/23) of postmortem isolates, of which 2/3 of these isolates were MDR. The virulence genes were not in accordance with the phenotype, as 2 out of 25 *rmpA* and *wzi-K1* positive isolates expressed the hypermucoviscous phenotype, and both were *wzi-K1* positive.

**Table 3:** Virulence factors of *Klebsiella* spp. recovered from children admitted to the Manhiça District Hospital (severe disease) and postmortem specimen from the Child Health and Mortality Prevention Surveillance

	Bacteremia *n =* 88 (%)	Postmortem *n =* 23 (%)	*P* value	All isolates *n =* 111 (%)
**Protectins/invasins**				
*rmpA*	8 (9.1)	7 (30.4)	0.011	15 (13.5)
*magA*	7 (8)	8 (34.7)	0.002	15 (13.5)
*traT*	9 (10.2)	14 (60.8)	< 0.0001	23 (20.7)
**Adhesins**				
*fimH*	30 (34.1)	2 (8.6)	0.021	32 (28.8)
*mrkD*	52 (59.1)	15 (65.2)	0.434	67 (57.7)
*ycfM*	43 (48.9)	15 (65.2)	0.105	58 (52.3)
**Toxin**				
*hylA*	13 (14.8)	2 (8.6)	0.731	15 (13.5)
*hylC*	8 (9.1)	0	0.354	8 (7.2)
**Siderophores**				
*iutA*	7 (8)	1 (4.3)	1	8 (7.2)
*fyuA*	5 (5.7)	0	0.581	5 (4.5)
*entB*	57 (67.9)	15 (65.2)	0.609	72 (64.9)
**SPATES**				
*Sat*	9 (10.2)	2 (8.6)	1	11 (9.9)
*sepA*	3 (3.4)	0	1	3 (2.7)
*piC*	2 (2.3)	2 (8.6)	0.178	2 (1.8)
*sigA*	3 (3.4)	1 (4.3)	1	4 (3.6)
*Pet*	10 (11.4)	4 (17.3)	0.472	14 (12.6)

The SPATE encoding genes, *sat, sepA, piC, pet* and *sigA* were detected in 9.9% (11/111), 2.7% (3/111), 1.8% (2/111), 12.6% (14/111) and 3.6% (4/111) of *Klebsiella* isolates, respectively, with similar distribution between postmortem isolates and isolates from admitted children (Table 3).

Among MDR isolates from admitted and post-mortem children, 73.3% (11/15) and 75.9% (44/58) carried siderophores genes, 73.3% (11/15) and 82.7% (48/58) carried adhesion genes, 80% (12/15) and 31% (18/58) carried invasins/protectins, 33.3% (5/15) and 24.1% (14/58) carried SPATES and 13.3% (2/15) and 22.4% (13/58) carried toxin genes, respectively. Almost all isolates from both admitted (100%, 7/7) and postmortem children (75%, 6/8) or (87.5%, 7/8 vs. 71.4, 5/7) carrying wzi-K1 and *rmpA* genes were MDR.

### Outcome of *Klebsiella* infections in bacteremic patients

Of the admitted children, outcome data was available for 75 out of 88 children (85.2%) with *Klebsiella* bacteremia, yielding an in-hospital case fatality rate (CFR) of 30.7% (23/75), which was particularly high (43.5%; 10/23) among younger than 2 years (between 12 and 23 months), and 11 children were transferred or left the hospital.

**Fig. 2:**
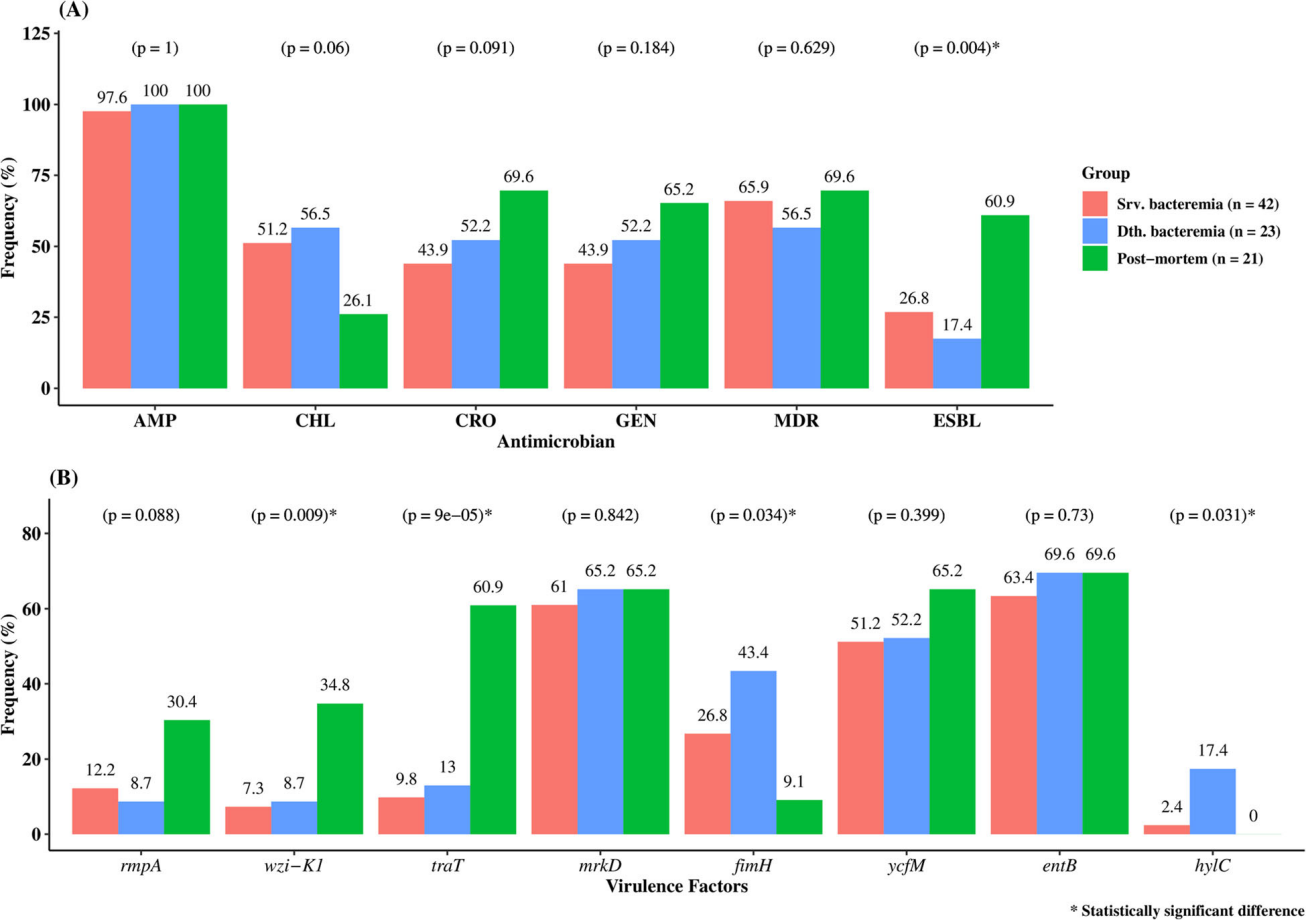
Comparisons between isolates from children with bacteremia who survived, who died and postmortem children included in the CHAMPS study for (**A**) non-susceptibility to antimicrobials used for empirical treatment (**B**) and the common virulence genes for each group

Resistance to chloramphenicol (56.5%, 13/23 vs. 51.2%, 21/41 *p =* 0.06) occurred more frequently among isolates from children who died when compared to those who recovered, as shown in Fig. 2A. Similarly, the postmortem isolates more often had a *wzi-K1, rmpA, traT* genes (Fig. 2B). Also, isolates from admitted children with poor outcome were likely to harbor *hylC* and *fimH* genes (Fig. 2A and B).

Isolates from admitted children who died were frequently ampicillin resistant (100%, 23/23), trimethoprim-sulfamethoxazole resistant (69.6%, 16/23), amoxicillin-clavulanic acid and chloramphenicol resistant (56.5%, 13/23), gentamicin and ceftriaxone resistant (52.2%, 12/23), or harboring *entB* (69.6%, 16/23), *mrkD* (65.2%, 15/23), or *ycfM* (52.2%, 12/23) genes. Almost all patients who died received oral aminoglycosides as empirical treatment, except three patients who took only cephalosporin, trimethoprim-sulfamethoxazole or chloramphenicol (Table 4). Furthermore, these patients died in the first 48 h (56.5%, 13/23) of hospitalization, except for those who had adequate antibiotherapy, but had an array of genes, including *rmpA*, *hylC*, *pic*, *saT*, *sepA* and *fimH*, who died after 6 days or more of hospitalization.

**Table 4:** Resistance and virulence profile of isolates from bacteremic children who died after admission

Isolate ID	Resistance profile	Virurence profile	Antibiotherapy	Co-infection	Days of hospitalization
392.5	AMP-AMK-AUG-CRO-CAZ-ATM-SXT-CHL-CXM	*fimH*-*mrkD*-*entB*-*bla*_*CTX-M-15*_	Unkown		2
034.5	AMP-AUG	*fimH*-*hylA*-*mrkD*-*ycfM*-*entB*	Unkown		1
8055	AMP-GEN-SXT	*ycfM*-*pet*-*entB*	Unkown		6
937.6	AMP-AUG-CRO-ATM-GEN-TOB-SXT-CHL-CXM	*mrkD*-*ycfM*-*entB*-*bla*_*TEM-1*_	Unkown		1
208.4	AMP-SXT	No virulence genes	Aminoglycosides-ampicillin/amoxicillin	*Streptococcus* group D	1
580.4	AMP-AUG-CRO-ATM-GEN-TOV-SXT-CL-CXM	*mrkD*-*ycfM*-*entB*-*wzi-K1*	Chloramphenicol		4
439.9	AMP-AUG-CRO-ATM-GEN-TOB-SXT-CHL-CXM	*mrkD*-*ycfM*-*entB*-*bla*_*SHV-1*_	Chloramphenicol- Aminoglycosides-ampicillin/amoxicillin	*Pseudomonas aeruginosa*	3
522.4	AMP	*fimH*-*hylAI-ycfM*-*sat*-*entB*	Aminoglycosides-ampicillin/amoxicillin	*Staphylococcus aureus*	1
563.7	AMP	*mrkD*-*ycfM-traT*	Chloramphenicol		2
676.9	AMP-AUG-CRO-ATM-GEN-SXT-CHL-CXM	*fimH-hylA-mrkD-sat-entB-trat-hylC-bla*_*TEM-1*_	Aminoglycosides		1
785.3	AMP-AMK-AUG-CRO-ATM-GEN-TOB-SXT-CHL-CXM	*mrkD-ycfM-sigA-entB-bla*_*CTX-M-15*_*-bla*_*SHV-1*_	Cotrimaxozol		3
520.4	AMP-AUG-CRO-GEN-SXT-CHL-CXM	*fimH-mrkD-ycfM-entB-bla*_*CTX-M-15-*_	Penicillin-aminoglycosides-cephalosporin		15
401.8	AMP	*fmH-mrkD*	Cotrimaxozol-aminoglycosides-ampicillin/amoxixillin-cephalosporin		1
009.1	AMP-AUG_CRO-CAZ-GEN-SXT-CHL-CXM	*hylA-mrkD-entB-traT-hylC-bla*_*CTX-M-15*_*-bla*_*SHV-1*_	Aminoglycoside-ampicillin/amoxicillin		1
073.2	AMP-AUG-CRO-ATM-GEN-SXT-CHL-CXM	*mrkD-ycfM-entB-rmpA*	Cotrimaxozol-aminoglycoside-ampicillin/amoxicillin-cephalosporin		18
334.2	AMP	*mrkD-ycfM*	Cotrimaxozol-chloramphenicol-aminoglycoside-ampicillin/amoxicillin		12
908.4	AMP-AUG-CAZ-GEN-TOB-SXT-CHL	*fimH-mrkD-saT-sepA-piC-entB-hylC*	Penicillin-cotrimaxozol-aminoglycoside		9
909.1	AMP-SXT	*entB*	Penicillin-aminoglicoside		4
9285	AMP-CRO-CAZ-ATM-GEN-NA-SXT-CHL-CXM	*entB- bla*_*SHV-1*_	Cotrimaxozol-aminoglycoside-ampicillin/amoxicillin-cephalosporin		3
657.9	AMP-AUG-CRO-CAZ-ATM-TOB-NA-CIP-SXT-CHL-CXM	*fimH-saT-peT- bla*_*CTX-M-231*_*-bla*_*SHV-1*_	Aminoglycoside-ampicillin/amoxicillin		1
143.8	AMP	*fimH*	Chloramphenicol-aminoglycoside-ampicillin/amoxicillin		1
522.4	AMP	*fimH-mrkD-entB*	Cephalosporin		6
218.4	AMP-AUG-CRO-ATM-GEN-TOB-SXT-CHL-CXM	*ycfM-saT-entB-hylC- bla*_*CTX-M-15*_	Aminoglycoside-ampicillin/amoxicillin		1

*AMP* Ampicillin, *AMK* Amikacin, *AUG* Amoxicillin-clavulanic acid, *TZP* Piperacillin-tazobactam, *CXM* Cefuroxime, *CRO* Ceftriaxone, *CAZ* Ceftazidime, *ATM* Aztreonam, *ETP* Ertapenem, *IPM* Imipenem, *MEM* Meropenem, *NA* Nalidixic acid, *CIP* Ciprofloxacin, *CHL* Chloramphenicol, *GEN* Gentamicin, *TOB* Tobramycin, *TET* Tetracycline, *SxT* Trimethoprim- Sulfamethoxazole, *MDR* Multi-drug resistant (defined when isolates are resistant ≥3 unrelated antibiotic groups), ESBL – Extended-spectrum β-lactamase phenotype

Among children with clinical information, 65.3% (49/75) carried MDR isolates, of which 26.5% (13/49) died. Additionally, 12 out of 75 carried isolates that were MDR and harbored SPATE genes, of which 50% of them died; and 40% of children carrying isolates that harbored toxin or invasins and protectin genes and were simultaneously MDR (15/75 and 10/75), died as well.

From the post-mortem children, clinical information was available for 21 out of 23 children, among which *Klebsiella* spp. was in the chain of event of death as the immediate cause of death in approximately one third of children; and in-hospital associated mortality was 95% (20/21), with an average of hospital admission stay of 4 days (Table S1). The clinical presentation of those patients was available in 18 out of 21 children, with difficulty of breathing (55.6%, 10/18) being the most common followed by fever (44.4%, 8/18), cough (38.9%, 7/18), loss of appetite (33.3%, 6/18) and diarrhea (27.8%, 5/18). Sepsis was the immediate cause of death for almost 43% (9/21) of post-mortem children, while respiratory infections, including pneumonia was the second major immediate cause of death among post-mortem children accounting for one third of the children. All post-mortem isolates were resistant to at least one antibiotic and 59% (13/22) were MDR, of which, 38% (5/13) carried *rmpA* gene and 23% (2/13) carried both *rmpA* and capsular serotype K1 gene (*wzi-K1*).

## Discussion

This is the first report comparing the antimicrobial susceptibility profile, its associated mechanisms of resistance, and the virulence repertoire between *Klebsiella* spp. isolates from postmortem studies and isolates recovered from children < 5 years of age with severe disease. The finding of a higher prevalence of MDR strains harboring both ESBL and genes associated with the hypermucoviscous phenotype in postmortem isolates may suggest the potential virulence and contribution of specific strains of *Klebsiella* to life-threatening infections, and thus, its potential role in childhood mortality in Mozambique.

The high CFR observed among children admitted with bacteremia may be explained by the high proportion (66%) of MDR strains. Most MDR isolates were also resistant to ceftriaxone due to the presence of *bla*_*CTX-M-15*_ in more than 25% of isolates limiting the therapeutic options, which is consistent with our previous observations of the poor outcome of *Klebsiella* associated bacteremia in children younger than 14 years of age [4, 22, 40]. The low resistance to chloramphenicol among postmortem isolates may be explained by the fact that this antibiotic was used as a drug of choice for empirical therapy [4] and is no longer a choice for routine clinical management. On the other hand, the presence of *hylC* and *sat* among the fatal strains from bacteremic children may also help to explain the *Klebsiella* associated mortality, as they have been described in *E.coli* showing cytotoxic effect on erythrocytes and epithelial cells, respectively, by disrupting the membrane or autophagy and cell detachment [41–43]. The excess of *Klebsiella* associated mortality is similar to previous studies showing mortality rates up to 38.5% in other countries [44, 45], including in the neighboring country, South Africa (26–35%) [46].

As previously reported in this community and elsewhere [10, 17, 22, 47, 48], an important public health concern is related to the emergence and widespread dissemination of ESBL-producing *Klebsiella* spp., challenging the appropriate management of MDR *Klebsiella* infections, that were mostly treated with ceftriaxone. The major dilemma is related to the finding of strains with reduced susceptibility to carbapenems (meropenem and ertapenem), often used as a second-line treatment options, as per international guidelines, but not foreseen in our setting [49]. This is alarming as these antibiotics are not routinely used in our study community and are rare in Mozambique in general, even though, the isolates remained susceptible to imipenem. Our findings provide a clear indication that an appropriate action plan is required to contain the spread of antibiotic resistance, mainly because the isolates remained susceptible to antibiotics that are expensive and unavailable in most of the rural communities, constraining their use even when the available antibiotherapy is ineffective [50].

The high frequency of genes involved in the synthesis of capsule (*rmpA* and *wzi-K1*), known to inhibit and evade phagocytosis by host cells and to neutralize the antibacterial activity of host defenses [13], in postmortem isolates may suggest potential virulence and fatality of the strains recovered in deceased children. The presence of these genes, mainly, *rmpA* give an indication that hipervirulent *Klebsiella* may be circulating in our setting, as this gene is one of the markers for hv*Kp* [8, 9, 51]. However, this should be interpreted with caution, as the other markers were not investigated. The *wzi-K1* gene indicate the circulation of the *K1* capsular serotype.

The high prevalence of siderophores was expected among pathogens causing invasive disease as these are essential for iron acquisition and survival of *Klebsiella* when interacting with host cells [12]; in addition to the high prevalence of adhesins that are critical for the first step of bacterial colonization /adhesion, with several variants that have been described [10, 11].

As of this writing, to our best knowledge this is the first report on the occurrence of the studied SPATEs in *Klebsiella* spp., suggesting that they potentially play an important role in the pathogenicity of *Klebsiella*, as previously described [52–54]. In addition, the sequences of SPATEs reported here are similar to those reported in other *Enterobacteriaceae* species as previously reported and may play the same role [52, 54].

There are some limitations in this study, one of these is that our study compared *Klebsiella* spp. in admitted patients and postmortem samples from different study periods; none of the children with bacteremic *Klebsiella* isolates was included in the postmortem study, even in the period when the IBD surveillance and CHAMPS overlapped. In deep molecular characterization including phylogenetic analysis, serotyping and multi-locus sequence typing are underway to better understand the molecular epidemiology of isolates from bacteremia and postmortem samples. This data cannot be generalized for the Mozambican or African population, as the samples were from only one geographic setting.

## Conclusion

Our data calls for the urgent need for more effective alternative antibiotics for management of fatal virulent MDR *bla*_*CTX-M-15*_ and *bla*_*SHV-1*_ *Klebsiella* strains and the implementation of appropriate measures to contain AMR (e.g. prudent use of antibiotics for empirical treatment, reduce the antibiotic usage in the agricultural activity, improve hospital infection control, rationalize the antibiotic use in the community). Continuous monitoring and surveillance for antimicrobial resistance (AMR) are crucial to support defining local and international guidelines for effective alternative options for anti-biotherapy or guide the design and development of new intervention tools (e.g. vaccines with broad coverage spectra), which requires the description of serotypes circulating.

## Supplementary Information

**Additional file 1: Table S1.** Clinical picture of post-mortem patients recruited in the CHAMPS Study.

## Data Availability

The datasets used and analysed during the current study are available from the corresponding author on reasonable request.

## Abbreviations

ESBL: Extended Spectrum Beta-Lactamase
MDR: Multidrug Resistance
AMP: Ampicillin
AMK: Amikacin
AUG: Amoxicillin-clavulanic acid
TZP: Piperacillin-tazobactam
CXM: Cefuroxime
CRO: Ceftriaxone
CAZ: Ceftazidime
ATM: Aztreonam
ETP: Ertapenem
IPM: Imipenem
MEM: Meropenem
NA: Nalidixic acid
CIP: Ciprofloxacin
CHL: Chloramphenicol
TOB: Tobramycin
GEN: Gentamicin
TET: Tetracycline
SXT: Trimethoprim-sulfamethoxazole
CISM: Centro de Investigação em Saúde de Manhiça
PCR: Polymerase Chain Reaction
hv*KP*: hypervirulent *Klebsiella pneumonia*
c*KP*: Classical *Klebsiella pneumonia*
MITS: Minimally Invasive Tissue Sampling
CHAMPS: Child Health and Mortality Prevention Surveillance
DSS: Demographic Surveillance System
MDH: Manhiça District Hospital
HDSS: Health Demographic Surveillance System
CSF: Cerebrospinal Fluid
CLSI: Clinical Laboratory Standard Institute
SPATE: Serine Protease Autotransporters of Enterobacteriaceae
IBD: Invasive Bacterial Surveillance
CFR: Case Fatality Rate
